# The link between periodontal disease and cardiovascular disease: How far we have come in last two decades ?

**DOI:** 10.4103/0972-124X.75908

**Published:** 2010

**Authors:** Prasad Dhadse, Deepti Gattani, Rohit Mishra

**Affiliations:** *Department of Periodontics, Hitkarini Dental College and Hospital, Dumna Road, Jabalpur - 482002, India*

**Keywords:** Atherosclerosis, coronary artery disease, periodontitis, risk factor, systematic review

## Abstract

Many epidemiological studies have investigated the relationship between periodontal disease (PD) and cardiovascular disease (CVD), but their results are heterogeneous. This review article is designed to update the potential association, that forms the basis of understanding for a (causal) role for PD to cardiovascular events; as reported by various observational (case-control, cohort, cross-sectional) studies, epidemiological and interventional studies, not considering the other number of systemic health outcomes like cerebrovascular disease, pregnancy complications, chronic obstructive pulmonary disease, diabetes mellitus complications, osteoporosis, etc. A brief overview has been included for atherosclerosis (ATH), its pathophysiology and the association of periodontal infections as a risk factor for causing ATH, which seems to be a rational one; as development of ATH involves a chronic low-grade inflammation and moreover, it has long been set up prior to development of ischemic heart disease and thus provides potential contributing mechanisms that ATH may contribute singly or in concert with other risk factors to develop ischemic heart disease. This article goes on to discuss the correlation of evidence that is gathered from many scientific studies showing either strong, modest, weak or even no links along with their critical analyses. Finally, this article summarizes the present status of the links that possibly exist between PD and its role as a risk factor in triggering cardiovascular events, in the fairly long journey for the last two decades.

## INTRODUCTION

Cardiovascular diseases (CVD), including acute myocardial infarction and angina pectoris are major health problems in developing countries, and are considered amongst most common medical problems in the general population.[1,2] Annual mortality from CVD is about 12 million cases per year and are responsible for 30% of all deaths in the United States.[3] Cardiovascular diseases are estimated to have led to 1.59 million deaths in India in year 2000 and this figure is projected to increase to 2.03 million for the year 2010.[4] The Framingham Heart Study revealed that for people who reach the age of 40, 49% of men and 32% for women show clinical manifestations of ischemic heart disease during their lifetime.[1]

Gingivitis associated with extensive plaque and calculus deposits are most prevalent, extensive and severe in developing countries and in population with limited access to health education and dental care.[5,6] Mild forms of periodontal disease (PD) affect 75% of adults in the United States, and more severe forms affect 20 to 30% of adults. Since PD is common in population, it may account for significant portion of proposed infection-associated risk for CVD.[7] This can be reasonably explained by concrete evidence for current research, which states that ATH is the main underlying vascular disease responsible for cardiovascular and cerebrovascular morbidity and mortality.[8]

Cardiovascular disease affects 43 million individuals in the United States with a marked increase in geriatric population. Since this population group is increasing in number and since more elderly individuals are dentate than in the past, there is also an increased incidence of PD in this patient group.[9] This paradoxical finding is applicable to global population subsequently involving the proportionate risk for CVD.

This evidence coupled with recent evidence of linking PD to coronary heart disease suggest the need to evaluate the extent to which the strength of this association has been established through several scientific studies in the last two decades.

## PATHOPHYSIOLOGY OF ATHEROSCLEROSIS

Atherosclerosis (ATH) is an insidious process that typically takes decades to worsen to the point of causing signs and symptoms. The term is derived from the Greek words for hardening (sclerosis) and gruel or the accumulation of lipid (athere). The process is localized to the inner wall of arteries with a predisposition to form at locations of “disturbed” blood flow, such as points where arteries branch.

Atherosclerosis lesions begin with deposition of lipoproteins in the intimal layer of the affected artery. The lipoprotein particles such as low-density lipoproteins (LDLs) then seem to permit the accumulation of monocytes and lymphocytes in the intimal layer.[10] Early in the formation of atherosclerotic plaques, circulating monocytes adhere to vascular endothelium. This adherence is mediated through several adhesion molecules on the endothelial cell surface, including intercellular adhesion molecule 1(ICAM-1), endothelial leukocyte adhesion molecule 1 (ELAM-1) and vascular cell adhesion molecule 1 (VCAM-1).[11,12] Activation of monocytes (macrophage) in the blood vessels leads to release of hydrolytic enzymes, cytokines, chemokines and growth factors, which induce further damage leading to focal necrosis. The monocytes recruitment from the blood stream occurs which pass through the endothelium into the blood vessels and differentiate into macrophages, which slowly become lipid-laden “foam cells” characteristic of atheromatous plaques.[10,13] Macrophages also accumulate lipids especially LDLs in both oxidized and modified form. Modified LDL can be a major cause of injury to both endothelium and underlying smooth muscles.[9] These lipid-laden cells eventually die and leave a necrotic lipid-rich element behind, in the arterial wall. These lipid containing area calcify to varying degrees. At the same time, smooth muscle cells in the arterial wall are stimulated to migrate in the intimal layer, where they can proliferate.[10]

Meanwhile microvessels invade the affected area, which can cause intraplaque hemorrhages. A fibrous cap that faces the interior of the artery eventually covers the atherosclerotic lesion.[10] A fatty streak can become a fibrous plaque, which becomes complex with lipid core, calcification and deposition of extracellular matrix proteins. Activated T-cells may stimulate metalloproteinase production by macrophages; which remodel the fibrotic plaque. Through remodeling of the extracellular matrix, the fibrous cap may become thin and rupture leading to activation of clotting system with thrombosis and subsequent occlusion of the artery that may be responsible for as many as one half of the cases of myocardial infarction.[14]

The role of infections has been discussed for many years. Recently, evidence has shown that certain common oral infections play a significant role in ATH[9] Atherosclerosis can occur in large and medium size elastic and muscular arteries. They can lead to ischemic lesions of brain, heart or extremities and can result in thrombosis and infarction of affected vessels, leading to death.[15]

Atherosclerosis is an insidious process, supported by considerable body of evidence that it is an inflammatory disease.[8] This hypothesis is also termed as the Ross’, “Response to Injury Hypothesis” of ATH, popularized by the pathologist Dr. Ross who proposed that the initial lesions result from injury to the endothelium and lead to chronic inflammatory process in the artery.[16] However, this hypothesis was first postulated in mid-1800 by European pathologists.

### Role of infections in endothelial injury

There is accumulating evidence of an association between some common infections of man and ATH. One possible mechanism is through endothelial injury by infectious agents, triggering in part; an inflammatory response seen in ATH. The role of infections has been recently reviewed by Danesh and colleagues; there is mounting evidence that infection by *Chlamydia pneumoniae, Helicobactor pylori*, Periodontal bacteria, and Cytomegalovirus are associated with heart disease.[13,17]

There is increasing amount of evidence that periodontal infections may directly contribute to the pathogenesis of ATH and thromboembolic events by providing repeated systemic challenges with liposaccharides and inflammatory cytokines.[5]

Herzberg and co-workers have reported that the *Streptococcus sanguis* and *Porphyromona gingivalis* have been shown to induce platelet aggregation and activation through the expression of collagen-like platelet aggregation-associated proteins. The aggregated proteins may play a role in atheroma formation and thromboembolic events.[18]

A recent study by Haraszthy *et al*. identified periodontal pathogens in human carotid atheromas (direct evidence). Fifty carotid atheromas obtained at endarterectomy were analyzed for the presence of bacterial 16S rDNA by PCR (polymerase chain reaction) using synthetic oligonucleotide probes specific for periodontal pathogens Aggregatibacter actinomyecetemcomitans, *Bacteriodes forsythus, P. gingivalis* and *P. intermedia*. Thirty percent of specimens were positive for *B. forsythus*; 26% for *P. gingivalis*, 18% for Aggregatibacter actinomyecetemcomitans, and 14% for *P. intermedia*.[19] Additional direct evidence comes from infections with *P. gingivalis* that contribute to systemic inflammation comes from animal studies (mice) shows calcification of aortic atherosclerotic plaque with exposure to *P. gingivalis* infection.[20] Increasing the length of exposure to the pathogens increases the amount of calcification. Moreover 44% of atheromas have one or more periopathogens.[19]

These and other studies suggest that periodontal pathogens may be present in atherosclerotic plaques, where like other infectious organisms periodontal pathogens too play a role in atherogenesis.

## STUDIES ESTABLISHING THE LINK BETWEEN PERIODONTAL DISEASE AND CARDIOVASCULAR DISEASE

### Case control studies

In 1989, Kimmo Mattila and his co-workers in Finland conducted two separate case control studies totaling 100 patients with acute myocardial infarction and they compared these patients with 102 control subjects selected from the community. A dental examination was performed on all the patients and a dental index was computed. In this original report, subjects with evidence of oral infection were 30% more likely to present with myocardial infarction as against subjects without oral infections.[21]

In a second case control report, Mattila and co-workers noted association between dental infections and degree of ATH. This study examined the same subjects as the first report with diagnostic coronary angiography. Accordingly the left main coronary artery, the circumflex artery, and the left anterior descending artery were assessed diagnostically and graded for the degree of occlusion on a 5-point scale. Again the total dental index score was used as a general score for dental caries, periapical lesions, and periodontal infections. In a multivariate analysis, significant associations were found between dental infections, age and triglycerides and severe coronary atheromatosis. These links remain significant even after adjusting for other known risk factors like total cholesterol, HDL, smoking, hypertension, socioeconomic status, and body mass index.[21] Mattila’s provocative findings generated a great deal of interest in the scientific community.

The authors postulated that bacterial infections have profound effect on endothelial cells, monocytes–macrophages, thrombocytes and blood coagulation and lipid metabolism; and concluded that dental infections are the only risk factor outside the scope of classic coronary risk factors, which have shown independent association with the severity of adult coronary ATH in their multivariate assessment. Continuing to monitor for myocardial infarction among the cases in these first case control reports, Mattila *et al*. presented Cox proportional hazard models further implicating dental infections as a significant risk factor for new cardiovascular events.[22]

## COHORT STUDIES

De Stefano and co-workers assessed the association between PD and CVD with National Health and Nutrition Examination survey (NHANES) I, which followed subjects for 14 years. This cohort study examined several potentially confounding variables including age, gender, race, education, marital status, systemic blood pressure, total cholesterol levels, body mass index, diabetes, physical activity, alcohol consumption, poverty and cigarette smoking. These investigators reported that among the 9760 subjects examined longitudinally, those with periodontitis has 25% increased risk of coronary heart disease related to those with minimal PD adjusted for the co-variables mentioned above. Interestingly, males younger than 50 years of age with periodontitis were 72% more likely to develop coronary heart disease compared to their periodontally healthy counterparts.[23]

Using data in the normative aging studies, Beck and co-workers evaluated 921 men aged between 21 and 80 years who were free of coronary heart disease at baseline. Over 18 years follow-up period, 207 men developed coronary heart disease, 59 died of coronary heart disease, and 40 had strokes. Odds ratio adjusted for age and established cardiovascular risks factors were 1.5, 1.9, and 2.8 for periodontal bone loss and total coronary heart disease, fatal coronary heart diseases and stroke, respectively. These data indicated that persons with radiographic evidence of periodontitis were 0.5–2.8 times more likely to develop coronary heart disease or suffer from a vascular event.[24]

In a larger six-year cohort study, Joshipura and co-workers studied 44, 119 men in the health professionals via mailed questionnaire with a self-reported history of PD and missing teeth. This study found no significant relation between self-reported history of PD and incidence of heart disease after adjusting for traditional risk factors (RR - 1.04). The study did however demonstrate that men with tooth loss and PD were 70% more likely to exhibit coronary heart disease.[25]

Genco and co-workers investigated the association between periodontal infections and risk of CVD in 1372 in Native Americans of Gila River Indian community, a group with high prevalence of diabetes mellitus. At baseline, alveolar bone level was measured and cardiovascular status was monitored for up to 10 years for electrocardiographic evidence of CVD using a pooling criteria. Among all age groups alveolar bone level was predictive for coronary heart disease, but did not remain significant in a multivariate analysis.[RR - 2.68 (95% CI 1.30–5.50)]. In contrast, for persons younger than 60 years of age, alveolar bone level was predictive of coronary heart disease (odds ratio of 2.68).[26]

## CROSS-SECTIONAL STUDIES

Arbes and colleagues[27] evaluated the link between PD and CHD in the NHANES III, and found that the odds of having history of heart attack increased with the severity of PD. The highest severity of PD in the population was associated with the odds ratio (OR) for 3.8 [95% CI (1.5–9.7)] compared with no PD; after adjusting for age, sex, race, poverty, smoking, diabetes, hypertension, BMI, and serum cholesterol level. Thus this cross-sectional study confirmed the association and also showed a direct relationship between heart disease and increasing levels of PD.

Genco and colleagues[28] assessed the association between specific subgingival periodontal organisms and MI. They compared 97 subjects with non-fatal MI with 233 control subjects. A panel of nine subgingival bacteria was evaluated, and subjects infected with one or more of these bacteria were compared with non-infected subjects. For MI the adjusted OR (95% C.I) was 2.99 (1.40–2.65) for the presence of *B. forsythus*, and 2.52 (1.35–4.70) for *P. gingivalis*; two periodontopathic bacteria. These findings support the notion that specific pathogenic bacteria found in cases of PD also may be associated with myocardial infarction.

## META-ANALYSIS OF OBSERVATIONAL STUDIES

Janket *et al*. performed a meta-analysis of nine cohort studies of PD as a risk factor for future cardiovascular and cerebrovascular events RR 1.19; (95% CI [1.08–1.32]) and found an overall 19% increased risk of such events in individuals with periodontitis.[33] The increase in risk was greater (44%) in people under age 65.

Scannapieco *et al*,[34] concluded in an extensive systematic review that a moderate degree of evidence exists to support an association between PD and ATH, MI and CVD, but that causality is unclear.

Results of another metal-analysis by Khader *et al*[35] combining six cohort and two cross-sectional studies are lower RR 1.15; (95% CI [1.06–1.25]).

In 2009, a more promising and extensive metal-analysis of observational studies was conducted by Alessandra Blaizot *et al*.[2] in Toulouse, France, to examine the association between exposure to periodontitis and CVDs. Studies published between 1989 and 2007 (nearing two decades) were retrieved by electronic and manual search from seven databases. The included articles reported the results from observational studies and assessed the link between periodontal exposure and CVDs as confirmed by one of the following criteria: diagnosed coronary artery disease, angina pectoris, myocardial infarction, mortality due to cardiac pathology. The study characteristics were abstracted by independent researchers following a standardized protocol. The MOOSE guidelines for meta-analysis for observational studies were followed.[36] From 215 epidemiological studies, 47 were observational; of which 29 articles could be combined by meta-analysis methodology. The pooled odds ratio calculated from 22 case control and cross-sectional studies was 2.35 (95% CI [1.87–2.96)]; *P*<0.0001). The risk of developing CVD was found to be significantly (34%) higher in subjects with PD compared to those without PD (pooled relative risk from seven cohort studies was 1.34 (95% CI [1.27–1.4], *P*<0.0001). This result shows that subjects with PD have higher odds and higher risk of developing CVD.[2]

## INTERVENTIONAL STUDIES

Noack and colleagues[37] demonstrated that C-reactive protein levels were highest in patients who were infected with periodontal pathogens where as CRP is an independent risk factor for CVD; however, detailed information is lacking about the mechanisms by which CRP participates in the pathogenesis of atheroma formation. C-reactive protein localizes the complement in human hearts during myocardial infarction, suggesting that CRP binds diseased muscle tissue, fixes complement and hence, triggers complement mediated inflammation that contributes to atheroma formation.[38]

Recently (i.e. 2010), Cesar de Oliveira and colleagues conducted Scottish Health Survey[39] to examine if self-reported tooth brushing behavior is associated with CVD and markers of inflammation (C-reactive protein) and coagulation (fibrinogen). The database of the study drew 11,869 men and women from the population living in households in Scotland into the study. The results showed that there were a total of 555 CVD events over an average of 8.1 (SD 3.4) years of follow-up, of which 170 were fatal. In about 74% (411) of CVD events, the principal diagnosis was coronary heart disease. Participants who reported poor oral hygiene (never/rarely brushed their teeth) had an increased risk of CVD events (HR 1.7; 95% CI 1.3–2.3; *P*<0.001) in a fully adjusted model. They also had increased concentration of both C-reactive protein (β 0.04, 0.01–0.08) and fibrinogen (0.08,-0.01–0.18).

Interventional studies conducted by Ebersole and colleagues[40] have shown that treating patients who have PD with scaling, root planing and flurbiprofen is associated with a trend towards reduced CRP levels one year after therapy.

There are also studies being designed to look at the effect of intervention on CVDs. David Paquette *et al*., along with colleagues at Boston University; SUNY – Buffalo, University of Maryland and Oregon Health Science University (OHSU), have initiated plans for the “Periodontal Intervention and Vascular Events” (PAVE) pilot trial. This proposed multicentre study hopes to ultimately design and conduct a large clinical trial on periodontal therapy in patients at risk of cardiovascular events.

## SUMMARY

Epidemiologic studies show conflicting relations between PD and CVDs. Some studies have reported that PD is significantly associated with CVD as a risk factor,[24] while others have failed to show such correlation.[41] Interventional studies trying to explain this relationship generally use C-reactive protein as a major cardiac outcome with statistical methods unsuitable for its skewed distribution.[42] For this reason the interpretation and use of these results are questionable.[2]

For a causal role, however, the size of how the risk increases is one of the most important criteria by which epidemiologists judge causality, and the reported PD–CHD associations fall well below the limits of what is considered to be convincing. If the nine cohort studies had identified association that fell above some generally accepted limit (for example >200%), the need for methodological rigor would have been less pressing. Under those circumstances, a more detailed adjustment of smoking or health awareness would have been unlikely to lead to different conclusion. However, since nine cohort studies considered for this review consistently identified small or no risk increase, methodological rigor is essential. Even small errors in the control of smoking history, health awareness or other lifestyle factors can induce biases that are substantially larger than the observed PD–CHD association.[43]

The potential role of PD for causal role to CVD can be explained in a way that, since oral and CVDs have many factors in common, it is important to rule out these as alternative explanations before interpreting the link as causal. The evidence support a moderate link but not a causal relationship between PD and coronary heart diseases.[7,31]

From these studies, by assessing the total coronary heart disease and fatal heart disease, it appears that the analyses were adjusted for many of the important risk factors, which are relevant to both PD and heart disease. It is of considerable interest that Beck and colleagues also found that the cumulative incidence of coronary heart disease increase with greater levels of age-adjusted alveolar bone loss at baseline, suggesting a dose response that is, the more PD at baseline, the greater is the cumulative incidence of coronary heart disease over time.[12]

DeStefano and colleagues[23] found that PD and poor oral hygiene may be an indicator or surrogate for lifestyle affecting personal hygiene and health care, and thus explains the relationship of PD and heart disease. Thus cumulative index for coronary heart disease argues against lifestyle as a simple explanation for this association.

Meta-analysis of longitudinal studies have shown that pre-existent PD; as determined by direct oral examination, independently conferred excess risk for increased morbidity and mortality due to CVD. The increase risk ranges from a modest 20% (OR of 1.2) to 180% (OR of 2.8).

A study by Genco and colleagues[28] demonstrated positive correlation between PD and coronary heart disease in a population that is largely nonsmoking.

Some studies include more than 1000 subjects and other more than 10000 subjects. Some extend over decades. Most of these studies began as CVD studies and have controlled for traditional risk factors; such as gender, smoking, body mass, lipid profile, exercise, familial history, socio-economic status, education, and other cardiovascular risk factors. Thus several criteria appear to be satisfied for establishing an association; multiple studies, large number of subjects, prospective cohort design, valid cardiovascular and oral examination data, and specificity by controlling for confounders and co-variables [Tables 1 and 2].

**Table 1:** Summary of association between oral conditions and CVD in six longitudinal studies with positive findings

Source, year, total no. of subjects	Country (follow-up period)	Exposure	Outcome	Measure of association	Adjusted for potential confounders
DeStefano and colleagues,[23] 1993, 9,760 (men and women)	United States (15 years)	Russel’s periodontal index	Admitted to hospital/death from CHD* (men < age 50 years)	RR#=1.2$ RR=1.7$	Smoking, hypertension, age, sex, triglycerides, SES, diabetes, serum lipids, BMI, Previous myocardial infarction
Mattila and colleagues,[22] 1995 214 (182 males, 32 females)	Finland (7 years)	Total dental index	New myocardial infarction or death from CHD	HR^+^=1.2$	Age, sex, race, education, poverty, marital status, SBP**, BMI, Cholesterol level, diabetes, physical activity, alcohol use, smoking
Joshipura and colleagues[25] 1996 44,119 (male health professionals)	United States (6 years)	Reported tooth loss due to periodontitis in men	Fatal and non-fatal myocardial infarction and sudden death	RR=1.7$	Age, BMI, exercise, smoking, alcohol use, vitamin E, family history of myocardial infarction before age 60 years
Beck and colleagues,[24] 1996 921 (men)	United States (18 years)	Whole-mouth bone level	New CHD Fatal CHD Stroke	OR++=1.5$ OR=1.9$ OR=2.8$	Age, sex, cholesterol level, smoking, diabetes, blood pressure, family history, education.
Morrison and colleagues[29] 1999	Canada (23 years)	Mild, severe gingivitis periodontitis	Fatal CHD and stroke	RR at age 35–69 years; mild gingivitis=3.6$; severe=6.9$; periodontitis=3.4$	Age, sex, cholesterol level, smoking, diabetes, hypertension, province of residence
Wu and colleagues, 2000[30] 9962 adults	United States (NHANES-I: 21 years)	Gingivitis and periodontitis (>4 mm pockets); edentulous by Russell’s periodontal index	Incident non-hemorrhagic stroke	RR: Gingivitis=1.2$; periodontitis=2.1$	Sex, age, race, education, poverty index, diabetes, HT, smoking status, alcohol use, BMI, cholesterol level, sample design

CHD^*^ = Coronary heart disease

RR^#^ = Relative risk; HR = Hazard Ratio

OR^++^ = Odds ratio

SBP^**^ = Systolic blood pressure

$ = Statistically significant adjusted measure of association; 6S, JADA, Vol133, June2002.

**Table 2:** Summary of association between oral conditions and CVD in three longitudinal studies with negative findings

Source, year, total no. of subjects	Country (follow-up period)	Exposure	Outcome	Measure of association	Adjusted for potential confounders
Joshipura and colleagues,[25] 1996 44,119 (male health professionals)	United States (6 years)	Reported history of PD in men	Fatal and non-fatal myocardial infarction and sudden death	RR*=1.04	Age, BMI**, exercise, smoking, alcohol consumption, vitamin E use, family history of MI before age 60 years
Hujoel and colleagues,[31] 2000 8032 dentate adults	United States (National Health and Nutrition Examination Survey I: 21 years)	Gingivitis and periodontitis (>1-mm pockets) by Russel’s periodontal index	Death or hospitalization due to CHD$ or revascularization	Gingivitis HR%=NS#; periodontitis HR=1.14	Age, age squared, sex, race, poverty index, marital status, education, marital status/sex+, log++ smoking duration, log height and weight log alcohol use per day, physical activity, nervous breakdown, sample design
Howell and colleagues,[32] 2001 22,0711 (U.S. male physicians)	United States (12.3 years)	Reported history of PD	Death due to CHD, non-fatal myocardial infarction or stroke	RR=1.13 (confidence limits: 0.99-1.28) adjusted for age and treatment; RR=1.01 (confidence limits: 0.88–1.15) fully adjusted	Age, aspirin and beta carotene treatment assignment, smoking, alcohol use, history of hypertension, BMI, history of diabetes, physical activity, parental history of MI, history of angina

RR^*^ = Relative risk

BMI^**^ = Body mass index

CHD $ = Coronary heart disease

HR^%^ = Hazard Ratio

NS^#^ = Not significant

+ Marital Status / Sex = Interaction between marital status and sex

++ Log = Logarithm; 17S, JADA, Vol133, June2002

However, the magnitude of risk is variable and appears modest in many studies. Modest degree of excess risk, such as odds ratios less than 2, may potentially be due to what epidemiologists refer to as residual confounding or that the potential existence of underlying risk factors were not fully considered, adjusted for or even measured. This issue, however is, a potential problem as there is always one more parameter that could be considered in any study design.

The report by Arbes[27] *et al*. analyzing the NHANES III data shows a strong association between history of myocardial infarction (a very robust and valid measure for CVD) and increasing PD severity in a dose response manner. The greater the PD, the greater the risk with odds ratio greater than 5 for the most of severe PD groups. This was present after adjustment of traditional risk factors for CVD. With odds ratio of this magnitude, the likelihood is lower than residual confounding is responsible for a spurious finding. Thus the epidemiologic link data are fairly strong.

## CONCLUSION

It is now clear from the epidemiologic studies that a potential link does exist between PD and CVD. Oral healthcare professionals can identify patients who are unaware of their risk of developing serious complications as a result of CVD and who are in need of medical intervention.

Prospective interventional studies are required to determine the exact link between PD and CVD as well as to evaluate whether periodontal treatment may reduce the risk of developing CVD. Some studies which are in progress to evaluate the moderation of vascular disease (ATH) owing to interventional periodontal therapy and the extent to which it (ATH) is responsible for triggering cardiovascular events. However, the challenge remains whether PD can be considered one amongst the traditional risk factors for CVD as the link established from different studies is not limited to a recent CVD. Overall, PD seems to be associated with no more than a modest increase (~20%) in cardiovascular risk in the general population.

As the ongoing studies report and confirm the strength of the association between PD and CVD, in the next two decades, the oral healthcare professionals and the medical professionals have to prepare for better planning of prevention programs. It seems from the scientific evidence gathered so far that interventional periodontal care remains invaluable not only for oral health but for general health as well.
